# Measuring Dispositional Flow: Validity and reliability of the Dispositional Flow State Scale 2, Italian version

**DOI:** 10.1371/journal.pone.0182201

**Published:** 2017-09-06

**Authors:** Eleonora F. M. Riva, Giuseppe Riva, Cosimo Talò, Marco Boffi, Nicola Rainisio, Linda Pola, Barbara Diana, Daniela Villani, Luca Argenton, Paolo Inghilleri

**Affiliations:** 1 Department of Cultural Heritage and Environment, Università degli Studi di Milano, Milano, Italy; 2 Department of Psychology, Catholic University of Milan, Milano, Italy; 3 Applied Technology for Neuro-Psychology Lab., Istituto Auxologico Italiano, Milano, Italy; 4 Department of History, Society and Human Studies, Università del Salento, Lecce, Italy; 5 Department of Educational Human Sciences, Università degli Studi di Milano-Bicocca, Milano, Italy; Tampereen Yliopisto, FINLAND

## Abstract

**Objective:**

The aim of this study is to evaluate the psychometric properties of the Italian version of the Dispositional Flow Scale-2 (DFS-2), for use with Italian adults, young adults and adolescents.

**Method:**

In accordance with the guidelines for test adaptation, the scale has been translated with the method of back translation. The understanding of the item has been checked according to the latest standards on the culturally sensitive translation. The scale thus produced was administered to 843 individuals (of which 60.69% female), between the ages of 15 and 74. The sample is balanced between workers and students. The main activities defined by the subjects allow the sample to be divided into three categories: students, workers, athletes (professionals and semi-professionals).

**Results:**

The confirmatory factor analysis, conducted using the Maximum Likelihood Estimator (MLM), showed acceptable fit indexes. Reliability and validity have been verified, and structural invariance has been verified on 6 categories of Flow experience and for 3 subsamples with different with different fields of action. Correlational analysis shows significant high values between the nine dimensions.

**Conclusions:**

Our data confirmed the validity and reliability of the Italian DFS-2 in measuring Flow experiences. The scale is reliable for use with Italian adults, young adults and adolescents. The Italian version of the scale is suitable for the evaluation of the subjective tendency to experience Flow trait characteristic in different contest, as sport, study and work.

## Introduction

The flow of consciousness is a universal experience, which can be understood and experienced in various social and cultural contexts and can be considered to be trans-generational [1] [2] and trans-cultural [3]. The flow experience is closely related to an action or activity, and has a relatively short duration. In order to occur, *several elements need to be simultaneously present and active*. These are: balance between challenges and skills, intrinsic motivation, clear goals, self-determination, concentration on the task, immediate feedback, lack of conscious control, altered perception of time (faster or slower), absence of anxiety, absence of boredom, and a positive affective state [4] [5] [6] [7] The first three elements are considered to be the main ones; the others cannot occur if the first three are not manifest. It is also important to stress that for *Balance between challenges and skills* is intended the balance *subjectively perceived* by the individual between their own personal skills and the demands of the environment [8].

By virtue of the emotional and cognitive benefits that the person senses clearly and immediately when in flow, optimal experience is actively sought after. In particular, when a person finds flow in one or more specific activity, they choose to repeat those activities frequently. Consequently, given that by constantly repeating an activity with maximum commitment, the individual acquires greater expertise in that field, the subject needs to search for opportunities of action that become increasingly more complex, so that he can continue to find a state of flow within the same activity. In this sense, by promoting this process of *psychological selection* [9], the state of flow favors individual development [10] [11]. On the other hand, a characteristic of flow experience is also that it is an *autotelic experience* [12], namely an experience or a situation that is phenomenologically positive, valid and rewarding in itself. The repeated experience of flow, then, in addition to encouraging increased competence within a specific field or a specific activity, favors the evolution of the individual, promoting the development of an *autotelic personality* [13] [14] [15] [16] [17] [18] [19] [20]. An autotelic person is an individual who has the ability to understand and abstract the characteristics of the activities connected to Flow that are intrinsic and activating specifically for themself. Starting from the characteristics identified, the autotelic person is able to look for other activities that have the same characteristics, in order to find more occasion in which experiencing flow and to maximize the opportunities to find satisfaction in life [8].

The ability to identify activities and situations in which flow can be found leads people to increase their experience both in terms of quality and variety. This ability becomes more and more important in modern society, which offers increasingly complex challenges. Everyone is constantly stimulated by demands, and it’s all the more necessary to carve out spaces for hobbies, leisure, family, friends, and, in general, to what gives meaning to life [21]. It becomes more important to develop the ability to select relationships, activities, desires and needs in a creative and evolutionary way. In this selection process the experience of flow has a very important role, because it directs people to the selection of activities, places and relationships among an increasingly wide and varied range [22]. For this reason, it is important, for researchers and applied psychologists, to have simple but detailed tools that measure flow without requiring an excessive amount of time to administer, making it possible to compare data related to different activities and collected in different contexts. These tools should also useable to use in batteries of tests to assess the impact of the experience of flow in relation to other psychological variables.

## Dimensions & measures of flow: The DFS-2

The first instruments developed for detecting and analyzing the flow experience were aimed at looking into the psychodynamic characteristics and the variability of the occurrences of this experience. In the initial stages of research, qualitative instruments were used, such as the *Flow Questionnaire* [23], which provided rich accounts of the flow experience, and which made it possible to define optimal experience based on its subjective and universal nature. Another instrument that has been central to the development of research on flow (and is still widely used) is the *Experience Sampling Method* (ESM) (for a review see [5]). ESM is a very interesting instrument because it allows for the repeated measurement of an individual’s activities and their related thoughts and psychological states in natural daily settings. However, ESM downside is that the activity must be interrupted to fill in the diary, and it has to be compiled in randomized moments across several days [24] [25] [26].

In 1996 Jackson & Marsh [27] made a first attempt to create a structured questionnaire that would solve some of the limitations of the existing instruments and could be used with a focal approach for research in specific activities. The Flow State Scale (FSS) questionnaire was designed to measure flow experience and define its specific characteristics in quantitative terms in relation to the nine dimension (D1 –D9) that have been identified by Csikszentmihalyi in 1990 [4]:

D1. *Balance between challenge and skills*: people perceive the situation as challenging and stimulating, and perceive that their resources are balanced and are adequate to the situation.
D2. *Union between conscience and action*: people feel totally involved in the action: automatisms permit the person to provide a more fluid performance, without neither the perception of intrusive thoughts nor the perception of effort.
D3. *Clear goals*: clear, defined and measurable objectives derive from coherent and non-contradictive information. This increases motivation and gives meaning to the experience.
D4. *Immediate and direct feedback*: during the activity people receive punctual and clear feedback from the situation, so that they are able to monitor steadily how they’re doing with their task.
D5. *Focus on task*. Attention is solely focused on the current task and there’s no space for any unnecessary information.
D6. *Sense of control*. People have the perception of a spontaneous and automatic control.
D7. *Loss of self-consciousness*. People perceive to be part of the task they are performing. Psychological energy is fully focused on action and people feel free to act and careless of other’s judgment. Furthermore, the feeling that one’s limits can be overcome increases the feeling of perceived self-efficacy.
D8. *Transformation of time*. The perception of the flow of time is altered: in some cases it feels faster, in others it is perceived as slowed down.
D9. *Autotelic experience*. This dimension concern the intrinsic satisfaction that the person feels in carrying out the task, regardless of the expected results and of the other possible motivation to act. Subjective satisfaction emerges from the execution of the task, without any need for an external reward.

The FSS has been constructed in order to assess the experience of flow in specific field of sports [27] [28], and it is administered at the end of a sports event or session, to reflect the experiences of flow in the activity that the person has just finished. To implement the quality of the instrument, Jackson and colleagues have also developed a second scale that, instead of assessing the experience of flow in the “here and now”, can assess the predisposition of athletes to find flow as a stable characteristic or trait of their personality, the Dispositional Flow Scale (DFS) Dispositional Flow Scale [29] [30]. The final versions of FSS-2 and DFS-2 have been published by Jackson, Martin, & Eklund in 2008 [26]. Both scales were validated in a long version as well as in a short version. The long version is made up of 36 items, divided equally into nine factors which correspond to the nine dimensions described by Csikszentmihalyi [4]. The short version is composed of 9 items, one for each dimension. FSS-2 and DFS-2.

Over the last 10 years the FSS-2 & the DFS-2 have been translated and validated in different languages, and applied in different cultural contexts, almost exclusively with samples from the sports sector [31] [32] [33] [25]. These studies demonstrated the cross-cultural validity of the scales, and their utility in the administration of test batteries. Some authors have also used the two scales, and particularly the DFS-2, with samples involved in other types of activity, such as study,outodoor leisure and internet gaming, demonstrating that the instrument can be extended to experiential situations other than sports. (i.e. [34]; [25]; [35]; [36]; [37]; [38]; [39]; [40]).

The purpose of the present study is to analyze the psychometric properties of the Italian version of DFS-2, in order to provide an instrument that can be useful both in cross-cultural research and in studies with Italian samples. Another aim of the research is to demonstrate that the DFS-2, which is usually used for research in sports psychology, is also suitable for use in other areas of research. For these reasons we were particularly meticulous in the translation of the items from English into Italian, and we used a large sample of athletes, college students and workers for the validation process.

## Method

### Participants

The sample consisted of 843 subjects (60.69% female), with an age between 15 and 74 years (Mean = 31.79, SD = 12.52). All the participants agreed to participate in the study voluntarily. As for the level of education, 41.50% of the sample were high school graduates and 41.02% had a university degree. In terms occupation, 59.47% were employed, and 39.21% were students.

The sample was collected in different contexts, in order to differentiate the sample and make it as representative as possible against the national standard. The sample was divided into three subcategories (students, workers and sports) that have been used, together with other variables, to verify the structural invariance of the questionnaire.

### Recruitment

The participants were recruited directly by the researchers, in different situations: for students during breaks between classes, in different universities and different degree programs, for the social workers and teachers in their workplace (schools, social services) and a small percentage (10%) on line through direct contact with researchers; as it regards the sports at the premises of the sports associations or at the end of public competitions. Inclusion criterion was a self-reported belonging to the categories identified. There was no criterion for exclusion from the sample. Were, however, they excluded those questionnaires that were incomplete and therefore could not be processed (15%). The research sample consists of those persons who have voluntarily completed the self-administered questionnaire in full. The total sample of surveys collected was 986. Of the people contacted about 30% (rough estimate) was not willing to participate. The number of people contacted in total is therefore approximately 1400.

### Ethics statement

The questionnaires are anonymous and self-reported, and individuals choose to participate voluntarily. Participants were aware that the data collected would be used in a scientific study. Participants could quit the study at any time they wished. Identifying information of the participants was kept at a different place than the dataset itself, under lock and key, by the main investigator.

To Italian national legislation is not necessary the evaluation of the ethics committee. Institutional review boards exempt researchers from submitting the research deign to the Ethics Committee: for such kind of research (self-report, with adults from a community-sample), at the time the study the approval by Ethics Committee was not required (S1 File). In any case the research was approved by the ethics committee of the Isitituto Auxologico Italiano (S2 File). The research is in line with the statements of the Helsinki Declaration: All participants signed informed consent and completed voluntarily and anonymously the questionnaire.

### Procedure

#### Translation of the questionnaire

Following the guidelines for test adaptation [41] [42] [43] and in agreement with the authors of the original scale, the scale was translated with the method of back translation. The numbers of the items that we indicate are the same as the original DFS-2—General [44] [26] (Copyright 2009 by S.A. Jackson). Particular efforts were devoted to avoid culture and context biases [45] [46] [47] [48].

### Instruments

To measure the degree of positivity, we used the Positivity scale developed by [49]. This scale (α = .86) is composed of eight items, scored on a 5-point Likert scale (from 1 = “strongly disagree” to 5 = “strongly agree”).

To measure Satisfaction with Life, we used the scale developed by Diner & al [50] (SWL). This scale (α = .81) is composed of five items (from 1 = “strongly disagree” to 5 = “strongly agree”).

To measure work engagement, we used the Utrecht Work Engagement Scale (UWES) proposed by Schaufeli & Bakker [51]. This scale (α = .94) is composed of seventeen items (ranging from 0 = “never” to 6 = “every day”).

To measure Flow Experience, we used DFS-2.

Finally, participants provided information on their age, gender and level of studies.

### Data analysis

Firstly, a confirmatory factor analysis (CFA) was performed, in order to evaluate the congruence of the factorial structure emerging in the validation process of the original scale [27] in the Italian version of the DFS-2. We used the following fit indices with the respective thresholds: (a) the chi-square (χ^2^) test. Non-significant χ^2^ values indicate an acceptable fit of the model, but it is almost always statistically significant. (b) The comparative fit index (CFI) [52]. Values of ≥ .90 indicate an acceptable fit and ≥ .95 and indicate an excellent fit. (c) The Tucker Lewis index (TLI). The thresholds are the same as CFI [53]. (d) The root mean square error of approximation (RMSEA). Values of ≤.05 and ≤ .01 indicate, respectively, good and excellent fit [54]. Furthermore, RMSEA can be evaluated in terms of probability, with a *p-value* less of .05 [55]. (e) The standardized root mean square residual (SRMR) [56]. Values of ≤ .08 demonstrate a good fit [55].

The reliability and convergent and discriminant validity of DFS-2 were tested by the following indices: (a) the Cronbach’s *α*. (b) The composite reliability (CR) that must be ≥ .70 for satisfactory reliability [57]. (c) For convergent validity, the average variance extracted (AVE) that must be ≥ .50 and below the CR. For discriminant validity, (d) the maximum shared squared variance (MSV) and (e) the average shared squared variance (ASV). Both must be lower than the AVE [58]. In addition, the risk of multi-collinearity among the DFS-2 factors was controlled by the variance inflation factor (VIF) [59] [60].

To evaluate the structural invariance of the questionnaire, we classified the episode that subjects spontaneously reported as an example of their flow experience through six categories: (1) Social and Relational (this includes relational or family activities, as well as work situations or sports, but with an emphasis on sharing or relationships, and volunteering); (2) Sports and Physical Activity (sports, competitions, exhibitions, training, activities such as a coaching or refereeing, dance); (3) Intellectual and Study (all study activities, including exams, degree, diploma, successes related to study. It also includes intellectual reflective activities, such as designing or programming); (4) Work (work activities of various kinds, except when the relational aspect is predominant); (5) Art and Creative Activities (producing art, music, cooking, photography, and other activities, even that require creative competence); (6) Experiential (including special or one-time travel experiences, listening to music or attending concerts, sex, prayer). This category also includes those who completed the questionnaire without specifying the kind of experience they were referring to.

The same analyzes were performed for the three subsamples: workers, athletes and students.

Furthermore, we calculated the correlations between the variables of the study.

## Results

### Confirmatory factor analyses

We tested, for the 36-item version, a model that considers nine first-order factors (Focus on Task, Challenge-Skill Balance, Sense of Control, Clear Goals, Merging of Action and Awareness, Unambiguous Feedback, Loss of Self-Consciousness, Autotelic Experience and Transformation of Time) and a single second-order factor. We used the maximum likelihood estimation (MLM). This version showed acceptable fit indexes (χ^2^ (842, 585) = 1334.85; *p* < .001; CFI = .95; TLI = .95; RMSEA = .04 (.03; .04), *p* > .05; SRMR = .06). S1 Fig. shows the parameters of the model. Our results reflects the structure identified by Jackson and Marsh [27].

No alternative model shows acceptable values. In particular, among the models that have been taken into account also in other validation of the translation of the DSF-2, the one with one first-order factor, finds these indexes: χ^2^ (843, 594) = 4966.37; *p* < .001; CFI = .61; TLI = .59; RMSEA = .10 (.10; .11), *p* < .001; SRMR = .10; while the one with nine first-order correlated factors gives the following results: χ^2^ (843, 558) = 1096.55; *p* < .001; CFI = .71; TLI = .70; RMSEA = .10 (.08; .11), *p* < .001; SRMR = .08.

### Reliability and validity analyses

Each factor sufficiently differed from the others (Table 1). Furthermore, α indices good reliability of measurements. No relevant multicollinearity among the nine first-order factors analyzed [61] was found.

**Table 1:** Reliability; convergent, discriminant and validity tests; collinearity statistics.

Factors	α	CR	AVE	MSV	ASV	VIF
CSB	.83	.85	.56	.39	.30	2.41
MAA	.73	.80	.52	.41	.29	1.50
CG	.82	.83	.54	.37	.27	2.08
UF	.84	.80	.51	.40	.33	2.29
CTH	.83	.80	.54	.31	.26	2.06
SC	.85	.84	.53	.36	.36	3.25
LSC	.82	.76	.50	.32	.26	1.33
TT	.82	.73	.49	.30	.27	1.30
AE	.85	.75	.51	.32	.30	1.56
Flow	.94	.82	.56	.38	.32	-

(Note: CSB = Challenge-Skill Balance, MAA = Merging of Action and Awareness, CG = Clear Goals, UF = Unambiguous Feedback, CTH = Concentration on the Task at Hand, SC = Sense of Control, LSC = Loss of Self-Consciousness, TT = Transformation of Time, AE = Autotelic Experience)

### Structural invariance

As previously mentioned, we have verified the structural invariance of the scale for the six categories of flow experience: Social and Relational; Sports and Physical Activity; Intellectual and Study; Work; Art and Creative Activities; Experiential. Table 2 shows the fit indixes of the sub-samples. All sub-samples show satisfactory indixes, except for Social and Relational episodes. The number of subjects (29) does not guarantee the reliability of this data.

**Table 2:** Structural invariance of the DFS-2.

Episode	N	χ^2^; df; *p*	CFI	TLI	RMSEA; C.I.; p	SRMR
Creative activities	54	892.59; 585; .00	.91	.90	.06;(.06 .08); .02	.09
Experiential	125	683.12; 585; .00	.95	.94	.04; (.03 .05); .95	.08
Intellectual / Study	98	805.38; 585; .00	.92	.92	.06; (.05 .07); .07	.08
Job	162	887.67; 585; .00	.93	.92	.05; (.05 .06); .03	.08
Social / Relational	29	2528.79; 585; .00	.79	.77	.35; (.33 .36); .00	.18
Physical activity	366	969.39; 585; .00	.92	.91	.04; (.03 .05); .99	.06
Subsample						
Workers	249	947.59; 585; .00	.95	.94	.05; (.04 .06); .26	.06
Athletes	385	900.68; 585; .00	.96	.95	.04; (.03 .04); 1.00	.06
Students	209	784.84; 585; .00	.94	.94	.04; (.03 .05); .97	.07

In addition, we tested the model for the three sub-samples: workers (249 ss, 29.5%), athletes (385 ss, 45.7%), and students (209 ss, 24.8%. In this case, too, the data shows an acceptable fit (Table 2).

### Correlation analyses

In Table 3 is shown the correlations between the nine dimensions of the DFS-2. All correlations are significant with high values, for example, between Sense of Control and Challenge-Skill Balance (*r* = .73); between Unambiguous Feedback and Sense of Control (*r* = .69), and with low values, for example, between Unambiguous Feedback and Transformation of Time (*r* = .14), Sense of Control and Transformation of Time (*r* = .17) and Clear Goals and Transformation of Time (*r* = .18).

**Table 3:** Correlation, mean and standard deviation of the DFS-2’s dimensions.

	CSB	MAA	CG	UF	CTH	SC	LSC	TT	AE
CSB	-								
MAA	.44*	-							
CG	.58*	.37*	-						
UF	.64*	.41*	.61*	-					
CTH	.55*	.35*	.59*	.56*	-				
SC	.73*	.48*	.64*	.69*	.65*	-			
LSC	.32*	.40*	.25*	.28*	.29*	.34*	-		
TT	.19*	.30*	.18*	.14*	.23*	.17*	.29*	-	
AE	.43*	.31*	.42*	.38*	.47*	.41*	.29*	.39*	-
Mean	15.38	13.82	15.92	14.63	15.79	15.14	13.58	15.19	17.04
Std.Dev.	2.74	3.16	2.92	3.02	3.06	2.75	4.28	3.54	2.85

* *p* < .01

Table 4 shows the correlations between the DFS-2 dimensions, age, gender, Satisfaction with Life scale, Positivity scale and Utrecht Work Engagement scale. To highlight the significant correlations, age is blandly correlated with Clear Goals (*r* = .11), Unambiguous Feedback (*r* = .19), Focus on Task (*r* = .11), Transformation of Time (*r* = -.15), Sense of Control (*r* = .14) and Autotelic Experience (*r* = -.12). Satisfaction with Life is correlated with all dimensions with *r* between .08 and .25. Furthermore, the Positivity scale is correlated with all dimensions with *r* between .08 and .31. Finally, Utrecht Work Engagement Scale is correlated with Focus on Task (r = .29), Clear Goals (*r* = .23), Unambiguous Feedback (*r* = .18), Sense of Control (*r* = .18), Autotelic Experience (*r* = -.17), Transformation of Time (*r* = -.11) and Challenge-Skill Balance (*r* = .17). The correlation between UWES and the total score of DFS-2 is *r* = .23.

**Table 4:** Correlations between the DFS’s dimensions, gender (0 = female; 1 = male), age, satisfaction with Life scale, Positivity scale and Utrecht Work Engagement Scale.

	Gender	Age	SatLife	Positivity	UWES
CSB	.04	.07	.21**	.28**	.17*
MAA	.13**	-.06	.11**	.13**	.05
CG	-.02	.11**	.18**	.26**	.23**
UF	.04	.19**	.15**	.23**	.18**
CTH	-.05	.11**	.21**	.26**	.29**
SC	.01	.14**	.18**	.26**	.18*
LSC	.17**	.07	.17**	.19**	.11
TT	.00	-.15**	.08*	.08*	.17*
AE	.06	-.12**	.23**	.28**	.17*
Flow	.07	.06	.25**	.31**	.23**

* *p* < .05;

** *p* < .01

## Discussion

The primary purpose of this research were to evaluate the factorial validity and reliability of the Italian version of the DFS-2, to be used with Italian adults and adolescents, and to verify its applicability beyond the field of sports.

The analysis confirms the validity of the structure we hypothesized: nine first-order factors (Clear Goals, Challenge-Skill Balance, Unambiguous Feedback, Merging of Action and Awareness, Sense of Control, Focus on Task, Loss of Self-Consciousness, Autotelic Experience and Transformation of Time) and one second-order factor (Flow); however, neither alternative model, which consider only one first-order factor or only nine first-order factors, is consistent. The scale is reliable, as there is both sufficient differentiation and sufficient balance among the nine factors. This data is in line with the factor structure found by Jackson and other colleagues [26] [27]. The Italian scale is thus one of the most correspondent to the original version in English, along with the Spanish version [32], while several other validated translations, though they do not reflect the original structure, do show that a structure with only nine first-order factors is stronger, or in some cases equivalent, to the one with the two levels of factors [25] [31] [33].

The structural invariance of the questionnaire was demonstrated by considering both the situations that the participants identified as activating flow, and three subsamples. The situations were divided into six categories: Social and Relational; Sports and Physical Activity; Intellectual and Study; Job; Art and Creative Activities; Experiential Situation. In line with the literature, this data demonstrates that optimal experience can be experienced in situations of each kind. [1] [2] [4] [6]. The sample of Social and Relational experiences is not numerically sufficient to guarantee the reliability of this category, but in literature there is no data that confirms the fact that there is a different incidence of flow in socio-relational situations compared to individual experiences [5] [62]. On the contrary, literature on the topic proposes the field of social relationships as full of relevant factors that promote optimal experience, in connection with the subjective and cultural interpretation that individuals give to the socio-relational situation itself (see [63]). The subsamples we selected for administering the questionnaire were sports, students and workers. Athletes (professionals and semi-professionals) are the type of subject that has been most frequently used in the validation of the scale in the literature, and we therefore wanted to select a portion of the sample using the same parameters. University students are among the most commonly used sample in research in psychology, and have often been used in the validation of articles on the flow scales, so we wanted to add this to the previous subsample. Workers are among the categories less present in quantitative research on optimal experience, but they cover an area of increasing interest for researchers of the flow [64]. We have therefore chosen to use a third sub-sample of workers, identifying the subjects among social workers (psychologists, psychotherapists, educators and teachers), which in literature are considered to be professions chosen in part because they are a possible source of positive psychological experience [65] [66] This has also enabled us to maintain a sufficiently uniform sub-sample. The resulting data shows, first, that you can experience flow through any type of experience. Although for some situations, such as sports or creative activities, it is easier to identify optimal experience because of their specific characteristics, the data does not show a significantly greater or better flow experience in sports or creative activities compared to categories selected by the subjects. Secondly, structural invariance of the questionnaire shows that the DFS-2 Italian version is a valid and reliable instrument for measuring the experience of flow not only in sports, but also in situations and activities of various kinds, such as study, work, leisure and creative activities, and not only with athletes or students but also with the workers’ sample. Therefore we can assume that it is functional also with other subsamples, or that it can be extended to the Italian population at large.

The correlations between the nine dimensions of the DFS-2 are all significant with high values. This consistency was not found for all the validations of translated versions. In particular, in the validation of Portuguese and French versions, Loss of Self-Consciousness and Time Transformation were weak [31] [33]. This data is consistent with qualitative studies conducted with samples of athletes (e.g. [67] [68]), which show how these two dimensions are negatively correlated with the specific characteristics of sport. The Italian sample was composed of athletes (professionals and semi-professionals; 43.4%), university students and social workers (teachers, psychologists, educators), so the incidence of the intrinsic characteristics of sport may have influenced to a lesser extent the results of the statistical. This finding highlights the significance of Loss of Self-Consciousness and Transformation of Time as intrinsic characteristics of the flow experience, in accordance with relevant literature [4] [5] and it draws attention to the need to work with a more varied sample of situations and experiences in order to properly define and evaluate psychological constructs such as that of optimal experience, that may occur in trans-disciplinary situations and contexts.

Although the correlations between the nine dimensions of the DFS-2 are all significant and with high values, the values are not completely homogeneous. Particularly high values were found in the dimensions of Unambiguous Feedback, Sense of Control and Challenge-Skill Balance, while lower values are related to the dimension of Transformation of Time. Such a divergence can be explained by the impact of athletes and the sports sample, for whom the Transformation of Time dimension is not significant. One hypothesis is that Challenge-Skill Balance, Clear Goals and Unambiguous Feedback are preconditions of flow [69] and for this reason they emerge with greater predominance, especially in a large sample which refers to various experiences and situations in which flow can be found. Another hypothesis to justify the variation of the correlation score is that Challenge-Skills Balance, Intrinsic Motivation and Self-Determination are core/primary characteristics [8]. According to this interpretation, Sense of Control, Unambiguous Feedback and Clear Goals are central elements that enable the individual to feel Self-Determination and Intrinsic Motivation, and therefore they collect the highest correlation values, along with Challenge-Skills Balance.

In line with the literature [5] no correlation emerges between the nine dimensions of the optimal experience and gender, and there are only low correlations between age and the six dimensions. In this respect, age can effect these dimensions: more experience can be linked to older age, as for example Clear Goals and Sense of Control, or greater reactivity can be linked to younger ages, as for the dimension of Concentration on the Task.

There is an excellent level of correlation between the DFS-2 and each one of the nine dimensions and the positivity scale, as well as with the SWLS. The Positivity scale is designed to measure “Positivity”, defined as «the tendency to view life and experiences with a positive outlook» [49] (p. 702). Measuring the individual's attitude both towards their lives in general and towards the concrete experiences and sequence of events in life, this scale assesses, in fact, some factors that can be considered to be important correlates of flow, or even its preconditions. This affirmation is in line with the fact that the Positivity scale has the highest correlation score with the DFS-2 as a whole. In addition, the highest scores are found with the dimensions of Autotelic Experience, Clear Goals, Challenge-Skill Balance, Sense of Control and Concentration on the Task at Hand, which are the ones that can be most affected by a positive view of life and an optimistic approach to the experiences that everyday life offers.

The Satisfaction with Life Scale is considered to be one of the factors of the more general construct of Well-Being [50]. One of the most widely accepted definitions of well-being in the literature defines it as the result of three components: positive affect, negative affect and Satisfaction with Life [70]. In this view, Satisfaction with Life refers to the cognitive aspect of Well-Being. The SWLS relies thus on the Life-Satisfaction concept, considered to be an outcome of an evaluation of all the aspects of one’s existence, and maintained using personal criteria [71]. The subjects, in formulating the answer, evaluate their perceived life circumstances on a standard that they establish and which they deem to be appropriate for themselves; the level of satisfaction is therefore higher when living conditions come closer to the standard established. The focus on overall satisfaction allows subjects to evaluate the areas of their life based on their values, policies and standards. To assess the positive affect and negative affect factors of the Well-Being construct, researchers have used other tools, such as the Positive Affect and Negative Affect Scale (PANAS) [72]. Being the SWLS an assessment tool focused on cognitive aspects of the experience, it fits well with the DFS-2, in which the perceptual and cognitive dimensions far exceed the emotional ones. Just like for the positivity scales, the highest correlation for the SWLS is obtained with the general construct of the flow. This allows us to assume that Satisfaction with Life can be an important correlate, or even a predictor, of an individual's ability to identify situations sought to produce optimal experience [73], although with a slightly lower influence than Positivity. This assumption is supported by the high correlation between SRLS and Autotelic Experience. The other aspects that strongly correlate with Satisfaction with Life are Concentration on the Task and Challenge-Skill Balance, which are also those that require greater cognitive activity.

The Utrecht Work Engagement Scale shifts a little from the frame of the other scales used, in that it does not come under positive psychology. It assesses Work Engagement, defined as «a positive, fulfilling, work-related state of mind that is characterized by vigor, dedication, and absorption. » [51] (pp. 4–5) [74] (p 720). Differently from the concept of flow, that refers to a momentary and specific state, the concept of work engagement regards a more persistent and pervasive affective-cognitive state. This state is not focused on any particular object, event, individual, or behavior, but it is a state that depends on the interaction between the individual characteristics of the person and the characteristics of context and work activities. Again, this instrument measures an attitude, an individual's predisposition that is stable and not situational. Nevertheless, Work Engagement is a psychological state that, similar to the flow, is related to an action or behavior, albeit indirectly, in that it is related to the events and relationships that are encountered in the workplace. Similarly to Optimal Experience, Work Engagement has both a cognitive and an affective component, and it calls for perception of challenge, ability to persevere, strong cognitive, emotional and motivational involvement, and full concentration on the task. There is a good correlation between UWES and the total score of DFS-2, and the Work Engagement scale correlates positively with seven of the nine dimensions of the flow, with particular regards to Unambiguous Feedback, Clear Goal, Concentration on the Task at Hand and Sense of Control. These are all characteristics linked to perceived competence, defined by some authors [33] as one of the important correlates of flow, together with the autotelic personality, which also has a good correlation with the UWES, and intrinsic motivation.

## Conclusions

The Italian version of DFS-2 has proven to be valid and reliable, and its applicability to heterogeneous samples of subjects and different situations and experiences, including but not limited to sports, has been verified.

This questionnaire can easily be used in cross-cultural research, as it uses the same items as the original version in English, which has been translated and validated in different languages. The process of translation and adaptation to the Italian cultural and linguistic context has not changed the semantic correspondence of individual items, but has indeed allowed to make sentences clearer [47] [48], and this has certainly favored the quality of the results obtained from the validation sample.

The Italian version of DFS-2 can be very useful in research on the relationship between optimal experience and other closely related psychological variables, or in research on the relationship between flow and psychological dynamics closely linked to the activity selected as a source of flow (such as motivation, perceived competence, goal attainment—see for example [31] [32] [33]).

## Supporting information

S1 Fig Model of the Dispositional Flow Scale. (DOCX)

S1 File Codice etico AIP. Italian Ethics Code for Psychology Research.
(PDF)

S2 File Letter of approval of research design by Istituto Auxologico Italiano. (PDF)
