# Supplementary material for: Measuring Dispositional Flow: Validity and reliability of the Dispositional Flow State Scale 2, Italian version
Source: PLoS One. 2017 Sep 6;12(9):e0182201. doi: 10.1371/journal.pone.0182201 (PMC5587230; doi:10.1371/journal.pone.0182201)
Supplement: S1 Fig — (DOCX) [file pone.0182201.s001.docx]

**Tables &Figures**

Figure 1 – Model of the Dispositional Flow Scale

Item 1

Item 28

Item 10

Item 19

.52*

.45*

.35*

.23*

.43*

.80*

.74*

.75*

.69*

Item 2

Item 11

Item 20

Item 29

.64*

.39*

.29*

.67*

.64*

.84*

.78*

.60*

.60*

Item 3

Item 12

Item 21

Item 30

.54*

.40*

.47*

.33*

.41*

.73*

.77*

.77*

.68*

Item 4

Item 13

Item 22

Item 31

.51*

.37*

.38*

.27*

.41*

.79*

.79*

.77*

.70*

Item 14

Item 5

Item 23

Item 32

.62*

.35*

.33*

.39*

.45*

.82*

.81*

.74*

.62*

Item 6

Item 15

Item 24

Item 33

.48*

.34*

.42*

.09*

.44*

.76*

.81*

.75*

.73*

Item 7

Item 16

Item 25

Item 34

.36*

.55*

.24*

.79*

.24*

.85*

.68*

.87*

.80*

Item 8

Item 17

Item 26

Item 35

.39*

.64*

.43*

.92*

.29*

.76*

.60*

.84*

.78*

Item 9

Item 18

Item 27

Item 36

.49*

.41*

.33*

.67*

.40*

.82*

.77*

.77*

.72*

.87*

.57*

.82*

.85*

.78*

.45*

.95*

.27*

.57*

* p < .01

Note: The numbers of the items are the same as the original scale DFS-2 - General by Jackson, Martin and Ecklund (2008; 2010 Copyright © 2009 by S.A. Jackson).
