# Supplementary material for: Measuring Dispositional Flow: Validity and reliability of the Dispositional Flow State Scale 2, Italian version
Source: PLoS One. 2017 Sep 6;12(9):e0182201. doi: 10.1371/journal.pone.0182201 (PMC5587230; doi:10.1371/journal.pone.0182201)
Supplement: S1 File — Italian Ethics Code for Psychology Research. (PDF) [file pone.0182201.s002.pdf]

Codice etico AIP

Premessa

Questo Codice fa parte integrante del Regolamento dell'AIP e si propone di regolamentare gli aspetti etici dell'attività di ricerca e di insegnamento della psicologia. Le responsabilità di chi operi in psicologia in altro ambito (ad esempio quello libero professionale o sanitario pubblico) sono regolamentate dall'Ordine Professionale degli Psicologi alla cui normativa rimandiamo.

Sono qui presi in considerazione solo gli aspetti generali della ricerca e dell'insegnamento in psicologia. Esso verrà integrato da codici specifici concernenti la ricerca con persone in età evolutiva, con persone disabili, con animali, nonché gli strumenti di valutazione e i test. Tali codici specifici, collegati al Codice generale che qui presentiamo, potranno essere elaborati delle sezioni dell'AIP interessate direttamente al problema, qualora esse esistano e intendano farlo.

PRINCIPI GENERALI

Competenza

Chi svolge ricerca in psicologia deve essere consapevole delle proprie competenze e dei limiti di queste e usare solo quei metodi e tecniche per le quali abbia una adeguata preparazione scientifica e una corrispondente esperienza pratica. Deve inoltre aggiornarsi sulle teorie e le tecniche inerenti al proprio ambito di ricerca.

Nelle ricerche con persone o animali in cui sia possibile ipotizzare un potenziale danno fisico e/o psicologico (si veda il punto 3), devono essere comunque incluse nel gruppo di ricerca, o consultate, persone esperte dello specifico settore di indagine (per esempio di psicologia clinica, di neonatologia, di psicologia animale, e così via). In ogni caso, la preparazione di coloro che svolgono ricerca psicologica deve comunque essere tale da includere anche la capacità di evitare rischi per il benessere fisico e psicologico dei/delle partecipanti alla ricerca, siano questi persone o animali.

Integrità

Chi fa ricerca si deve qualificare esattamente per quanto riguarda i titoli di studio e professionali, la formazione culturale e le esperienze professionali precedenti, e l'attuale attività scientifica, di insegnamento e professionale. Le competenze devono essere rese esplicite in modo corretto alle persone che collaborano alla ricerca, o che vi partecipano, e in generale alle persone con cui si viene a contatto.

Responsabilità sociale

Le responsabilità primarie di chi svolge attività di ricerca includono i seguenti aspetti:

• impegnarsi a rispettare, e a fare rispettare, le norme di legge vigenti in materia di sicurezza, di sperimentazione e di ricerca con persone e animali;

• favorire la diffusione delle conoscenze allo scopo di aumentare il benessere della società e delle persone;

• fare il possibile perché sia evitato un cattivo uso delle ricerche, delle teorie su cui si basano e delle tecniche che utilizzano. Ciò comprende il fornire strumenti e insegnare tecniche in sedi non appropriate o a persone non sufficientemente preparate ad applicarle (si veda anche il punto 9);

• preoccuparsi dell'immagine che si dà della psicologia, sia in sedi scientifiche che attraverso i media (si veda anche il punto 8);

• preoccuparsi del benessere psicologico di tutte le persone con cui si lavora, e con cui a vario titolo si entri in contatto nelle diverse fasi della ricerca (ad esempio collaboratori/trici, studenti/esse e ogni tipo di personale in formazione).

NORME ETICHE

1. Consenso informato e libertà della persona di ritirarsi dalla ricerca

In tutti i casi in cui si vogliano utilizzare dati ottenuti in una ricerca (per esempio video o audioregistrazioni, risposte a questionari o a interviste, e così via), è necessario ottenere il consenso delle persone chi vi hanno partecipato, che devono, inoltre, essere informate in modo corretto e per loro comprensibile su tutti gli aspetti della ricerca che potrebbero indurle a ritirare il consenso. Deve anche essere chiaro il nome, l'eventuale istituzione di appartenenza e lo status scientifico e professionale di chi effettua la ricerca.

Chi partecipa alla ricerca deve essere esplicitamente informato della libertà di ritirarsi in ogni momento. La libertà di partecipare alla ricerca deve essere accertata con particolare cura nel caso di persone istituzionalizzate, ospedalizzate o detenute. Nel caso in cui vi sia una relazione esplicitamente asimmetrica fra chi partecipa e chi effettua l'indagine (ne è un caso la relazione docentestudente), è necessario evitare che il rifiuto di partecipare comporti esiti negativi (anche se si trattasse della semplice privazione dei vantaggi connessi all'accettazione come le diverse forme di *credit* che devono poter essere ottenuti anche con altre modalità).

A queste regole sono ammesse solo le seguenti tre eccezioni:

a) Quando una persona non sia in grado di esprimere il consenso, esso va richiesto a chi ne ha la responsabilità (per neonati/e e per bambini/e ai genitori; per scolari/e e studenti/esse, nel caso in cui la ricerca si svolga in ambiente scolastico, alle autorità scolastiche; per i soggetti con handicap psichico e in generale per i/le pazienti non in grado di dare il consenso, esso va chiesto a chi ne ha la responsabilità legale, e alle figure professionali che li abbiano in cura, siano esse di ambito medico o psicologico). Nel caso di minori in grado di comprendere la richiesta di collaborazione, occorre un doppio consenso: del/la minore e di chi ne ha la responsabilità.

b) Nel caso di ricerche svolte con metodi osservativi non intrusivi, in luoghi pubblici e senza la possibilità preventiva o successiva di contattare le persone, dunque in assenza di un loro consenso, ne va comunque tutelata la riservatezza, ad esempio rendendo non riconoscibili i volti e le voci al momento della diffusione dei risultati.

c) Per quanto riguarda le ricerche che prevedono l'uso dell'inganno, e quindi l'impossibilità di ottenere un consenso informato preventivo, si veda il punto successivo (2). Il consenso di chi partecipa deve essere ottenuto per iscritto nei casi in cui non sia tutelato l'anonimato e in tutti i casi

che implichino l'uso di procedure dolorose (si veda il punto 3) o potenzialmente disturbanti e di possibili violazioni della privacy.

2. Uso dell'inganno nella ricerca

Quando l'obiettivo scientifico lo richieda e non sia possibile usare metodi alternativi, chi partecipa ad una ricerca può essere tenuto all'oscuro o ingannato su alcuni aspetti della ricerca.

Chi conduce la ricerca deve comunque informare esaurientemente i/le partecipanti alla fine della prova o, in casi particolari, alla fine della raccolta dei dati, e ottenere il consenso informato all'utilizzazione dei dati stessi. Quando non sia possibile, per ragioni tecniche, fornire le informazioni immediatamente dopo la prova, occorre contattare la persona alla fine della ricerca per fornire, eventualmente anche per iscritto, una adeguata informazione.

Il colloquio di chiarimento e di rassicurazione alla fine della prova (o della ricerca) non deve riguardare solo la descrizione degli aspetti della ricerca su cui la persona è stata ingannata, ma deve anche proporsi i seguenti scopi:

a) ripristinare il suo stato di umore e di autostima precedente;

b) dare informazioni aggiuntive (anche estranee al progetto della ricerca) su aspetti di interesse del/della partecipante;

c) eliminare eventuali idee scorrette che la persona si sia fatta sulla ricerca o su se stessa, indipendentemente dalle richieste reali della ricerca.

Chi svolge la ricerca deve essere disponibile a rispondere alle richieste o ai dubbi che insorgessero anche in seguito. Questo si può ottenere permettendo ai/alle partecipanti di restare in contatto con chi ha condotto la ricerca, o predisponendo una breve lista di responsabili, all'interno della struttura di ricerca, a cui potrà rivolgersi anche in seguito.

3. Il rischio di danni permanenti o temporanei a chi partecipa alla ricerca

La ricerca non deve comportare alcun rischio di danni permanenti a chi vi partecipi, compresi effetti nocivi, anche a lunga scadenza, di agenti fisici e chimici.

Quando in una ricerca non si possa fare a meno di utilizzare tecniche che rendano possibili danni temporanei di carattere fisico o psicologico, per quanto minimi e differiti nel tempo, occorre fornire ampia informazione e ottenere un consenso scritto. Per danni temporanei si intendono sia quelli conseguenti alla somministrazione di stimoli anche minimamente dolorosi, sia quelli che procurano stati di disagio, sia l'invasione della sfera privata. Chi fa ricerca deve tenere conto anche dell'esistenza di differenze culturali e individuali.

Gli esperimenti in cui si prevede che i/le partecipanti siano sottoposti a stimoli dolorosi devono essere preceduti da prove che stabiliscano i livelli individuali accettabili di percezione del dolore nell'esperimento vero e proprio.

Anche nei casi in cui non siano normalmente prevedibili danni fisici o psicologici alla persona, chi fa ricerca deve mettere in atto tutte quelle procedure (per esempio un'intervista postsperimentale)

che permettano di accertare l'eventuale presenza di effetti disturbanti e, di conseguenza, di mettere in atto le procedure necessarie esplicitate nel punto precedente.

Particolare cura deve essere usata nei casi in cui la procedura di un esperimento implichi situazioni di disagio (per esempio relative all'ambiente, alla durata delle prove, alla qualità degli stimoli, a particolari posture) che possano essere disturbanti o addirittura pericolose per chi abbia problemi di salute. Nei casi dubbi, occorre preliminarmente intervistare la persona per accertarne lo stato di salute in riferimento ai rischi specifici implicati nella prova.

4. La riservatezza

A chi partecipa alla ricerca deve essere sempre garantita la possibilità dell'anonimato. Quando i dati vengano presentati in sedi scientifiche o in altri contesti, deve comunque essere garantita la non riconoscibilità personale di chi partecipa alla ricerca. Nei casi particolari in cui questo non sia possibile, deve essere ottenuto il consenso di chi abbia partecipato alla ricerca per quanto attiene ai prevedibili usi e alla diffusione dei dati che lo/la riguardano.

Nei casi in cui si usino metodi osservativi in cui le persone non siano informate preventivamente della loro partecipazione alla ricerca, occorre attenersi alle seguenti regole:

a) possono essere registrate (con sistemi audio, video, o semplicemente con appunti) solo le situazioni che si svolgano in un luogo pubblico;

b) non devono essere riconoscibili, a meno di un esplicito consenso scritto, coloro che partecipano alla ricerca.

Le possibili eccezioni alla tutela della riservatezza sono limitate ai seguenti casi:

a) la consultazione con altre figure professionali (ad esempio, in ambito medico o psicologico) tenute a loro volta ad analoga riservatezza;

b) la tutela della persona stessa, nel caso in cui si riscontri la necessità di fornire informazioni a una struttura sociale o sanitaria o all'autorità giudiziaria, nell'ambito dell'attuale legislazione in materia.

5. La protezione di coloro che partecipano alla ricerca

Chi svolge la ricerca è responsabile del trattamento ricevuto da coloro che vi partecipano (persone o animali che siano) da parte delle persone che collaborano ad essa (ad esempio, studenti/esse, laureandi/e, tirocinanti, tecnici di laboratorio o altre figure professionali). Occorre perciò accertarsi delle loro competenze relazionali e scientifiche per quanto riguarda l'ambito di ricerca in cui hanno contatti diretti con coloro che vi partecipano.

Qualora emergano dalla ricerca informazioni (anche estranee all'obiettivo proposto), su aspetti della salute fisica e mentale di coloro che vi partecipano che meriterebbero approfondimenti (ad esempio, deficit neurologici, problemi affettivi o cognitivi non diagnosticati in precedenza), occorre consigliare alla persona (o a chi ne ha la responsabilità legale) di rivolgersi a strutture che possano effettuare ulteriori indagini o possibili interventi.

6. Il trattamento del soggetto animale

Chi fa ricerca utilizzando animali è responsabile non solo del trattamento che essi ricevono durante gli esperimenti, ma anche del loro benessere fisico e psicologico nell'intero corso della ricerca. In particolare occorre tenere presenti i seguenti aspetti:

a) Il benessere dell'animale deve essere assicurato, anche al di fuori della sua prestazione sperimentale, quanto agli aspetti alimentari, igienici, abitativi e anche sociali.

b) Le competenze di tutti coloro che vengono a contatto con l'animale (dai problemi di pulizia e nutrimento a quelli più immediatamente connessi alla ricerca) devono essere adeguate al ruolo che essi hanno nel trattamento dell'animale. Le responsabilità di chi fa ricerca riguardano tutti gli aspetti e i momenti del trattamento dell'animale.

c) Le procedure dolorose, da mettere in atto solo quando non ci siano alternative ugualmente accettabili e quando siano giustificate dagli obiettivi della ricerca, devono essere il più possibile minimizzate. Questo vale anche per le situazioni in cui, anche in assenza di dolore fisico, gli animali siano sottoposti a stimoli emozionali negativi (come ansia o paura) e a situazioni di forte disagio e stress. Anche nel caso del soggetto animale, esperimenti che implichino dolore fisico devono essere preceduti da studi sulla tollerabilità degli stimoli dolorosi, allo scopo di stabilire preventivamente un limite.

d) Gli interventi chirurgici su animali, da realizzare solo quando non siano possibili sistemi alternativi, vanno sempre condotti in anestesia e in condizioni asettiche.

e) L'eliminazione dell'animale, quando questa si renda necessaria alla fine della ricerca, deve essere effettuata in modo rapido e indolore.

7. La diffusione delle ricerche in sedi scientifiche

In occasione della presentazione delle ricerche in sedi scientifiche (congressi, riviste, altre pubblicazioni), devono essere rispettati i seguenti requisiti:

a) Non devono essere presentati in alcuna sede, né pubblicati, dati falsificati, inventati o distorti in tutto o in parte. Non devono essere utilizzati dati raccolti in altre ricerche senza citare la fonte o avere il consenso di chi le ha condotte.

b) Coloro che hanno svolto la ricerca devono esplicitare il loro ruolo e la loro responsabilità riguardo ai dati raccolti. Alle persone che abbiano collaborato in modo sostanziale in varie fasi della ricerca va riconosciuta la contitolarità dell'articolo o della presentazione. Ad altre persone che abbiano avuto un ruolo meno sostanziale, ma riconoscibile, va fatto esplicitamente riferimento nel testo. Quando si utilizzi parte del lavoro di studenti/esse e di laureandi/e, il loro nome va citato come coautori o collaboratori o menzionato nei ringraziamenti, a seconda del lavoro effettivamente svolto.

c) Ogni presentazione di ricerche nelle sedi scientifiche dovrebbe avere un carattere di originalità e non essere la ripetizione di lavori già presentati. Nel caso si tratti di una ricerca già pubblicata o già presentata a congressi, ciò va dichiarato esplicitamente con gli opportuni riferimenti.

d) I riferimenti al lavoro altrui devono essere sempre espliciti e precisi. Ciò anche nel caso in cui non si tratti di ricerche già pubblicate, ma di materiale noto a chi scrive attraverso collaborazioni scientifiche e relazioni personali.

e) Chi fa ricerca in psicologia deve essere disponibile, per un periodo di tempo di tre anni dalla pubblicazione di una ricerca, a mostrare, su richiesta, i propri dati grezzi ad altri per scopi di discussione scientifica. In ogni caso va tutelata la riservatezza dei/delle partecipanti.

f) Nella comunicazione delle ricerche va evitato un uso del linguaggio che manifesti forme di discriminazione di genere, etniche, religiose o di qualsivoglia gruppo o minoranza.

8. La divulgazione attraverso i media

Coloro che svolgono ricerca in psicologia sono anche responsabili dei loro interventi personali relativi alla divulgazione della psicologia attraverso i mezzi di comunicazione di massa. Essi debbono essere improntati alle seguenti linee:

a) vanno presentate solo idee che siano sostenute da teorie adeguate, o almeno da una personale esperienza professionale. Non vanno presentati come definitivi dati preliminari per i quali non vi sia ancora un'adeguata certezza scientifica.

b) Occorre evitare di personalizzare il rapporto tra l'utente e chi svolge la ricerca psicologica (per esempio, somministrando test all'interno di spettacoli televisivi, o rispondendo a domande in trasmissioni radiofoniche o sui giornali). Inoltre occorre astenersi dall'esprimere valutazioni e giudizi su casi specifici che non siano basati sulla conoscenza diretta e personale del caso e su una adeguata documentazione.

c) Va assolutamente scoraggiato l'uso, anche parziale, di dati, tecniche o strumenti di indagine da parte di persone professionalmente non preparate a questo scopo (si veda anche il punto 9).

9. L'uso dei risultati della ricerca e l'insegnamento della psicologia

Chi fa ricerca in psicologia è impegnato a valutare l'adeguatezza delle sedi in cui presentare i risultati dell'attività di ricerca al fine di evitare fraintendimenti o un loro cattivo uso.

Occorre comunque evitare di insegnare l'uso di tecniche e di strumenti di ricerca in sedi non scientificamente appropriate. Particolare cura va posta nelle determinazione del livello di competenza personale necessario all' applicazione delle tecniche e degli strumenti di ricerca oggetto della didattica. Non deve comunque essere permesso alle persone in formazione un uso autonomo di tali strumenti, prima che queste abbiano raggiunto l'adeguata preparazione professionale.

Nell'insegnamento universitario e nella supervisione di ricerche altrui (come tesi di laurea, lavori di tirocinanti, lavori di collaboratori/trici in formazione) il/la docente è responsabile di tutti gli aspetti del trattamento del soggetto umano e animale. Devono essere messe in atto tutte le misure ragionevoli per accertare la competenza e l'adeguatezza dei collaboratori (come studenti/esse, tirocinanti, ricercatori/trici in formazione). Tra gli altri suoi compiti, deve accertarsi della correttezza con cui questi si qualificano all'esterno.

MODALITÀ E ORGANISMI DI CONTROLLO PER IL RISPETTO DEL CODICE ETICO

Coloro che violino le norme del Codice Etico nelle fasi della realizzazione della ricerca e della diffusione dei risultati possono incorrere in sanzioni che verranno decise dall'AIP e che potranno giungere fino all'espulsione. Chi venga a conoscenza di violazioni al Codice Deontologico Professionale da parte di colleghi o colleghe, ha l'obbligo di segnalazione all'Ordine Professionale Regionale o Provinciale degli Psicologi.

Si raccomanda l'istituzione di un Comitato Etico Locale presso ogni dipartimento, istituto e ente di ricerca. Essa avrà il compito di assicurare il rispetto del Codice Etico nelle ricerche svolte all'interno di tali istituzioni o con la loro collaborazione. Si suggerisce che la composizione della Commissione Etica Locale tenga conto delle varie aree disciplinari.

Nel caso di piani di ricerca proposti da studenti/esse, dottorandi/e, o altre figure che facciano parte dell'istituzione o anche che siano esterne ad essa, si suggerisce che venga sottoscritta una dichiarazione di presa d'atto del Codice Etico della Ricerca Psicologica e di impegno al suo rispetto. Chi propone la ricerca deve anche essere disponibile, su richiesta del Comitato Etico Locale, a fornire informazioni sui seguenti punti:

a) le tipologie dei partecipanti alla ricerca;

b) le modalità di richiesta del consenso informato; e, nel caso in cui non sia richiesto, le ragioni di tale scelta;

c) le modalità di svolgimento di eventuali colloqui di chiarimento e di rassicurazione;

d) l'uso eventuale di procedure dolorose o che diano disagio fisico;

e) l'uso eventuale di procedure che possano dare disagio psicologico;

f) il nome, la qualifica e l'esperienza di chi vi collabora, con una specificazione del ruolo svolto nella ricerca.

Il Comitato Etico Locale potrà approvare direttamente il progetto di ricerca presentato. Nel caso in cui la ricerca ricada in casi particolari (ad esempio, preveda procedure dolorose o l'uso dell'inganno), il Comitato Etico controllerà con particolare cura il rispetto delle garanzie richieste dal Codice Etico e dagli eventuali codici delle sezioni implicate. Qualora la decisione richieda una discussione più approfondita, il progetto di ricerca potrà essere inviato a un organismo nazionale (la Commissione Etica dell'AIP) che lo esaminerà e formulerà un parere. Tale commissione verrà nominata dal direttivo dell'AIP, tenendo presenti le diverse aree disciplinari.

Memoria AIP sul progetto di legge N. 5442
