# Supplementary material for: Measuring Dispositional Flow: Validity and reliability of the Dispositional Flow State Scale 2, Italian version
Source: PLoS One. 2017 Sep 6;12(9):e0182201. doi: 10.1371/journal.pone.0182201 (PMC5587230; doi:10.1371/journal.pone.0182201)
Supplement: S2 File — (PDF) [file pone.0182201.s003.pdf]

ISTITUTO AUXOLOGICO ITALIANO
ISTITUTO DI RICOVERO E CURA A CARATTERE SCIENTIFICO

To: **Whom it May Concern**

From: Prof. Andrea Gaggioli, Ph.D.
Head, Project Review Board (PRB)

July 19, 2016

Subject: Review form for Project: "Measuring Dispositional Flow: Validity and Reliability of the Italian Version of the Dispositional Flow State Scale 2"

Review Form

Project: **"Measuring Dispositional Flow: Validity and Reliability of the Italian Version of the Dispositional Flow State Scale 2"**

Leading Researcher/Supervisor:

Riva, E.

Other Researcher/s:

Riva ,G., Talò, C., Boffi, M., Rainisio, N., Pola, L., Diana, B., Villani D., Argenton, L., Inghilleri, P.

Date of application:

14 June 2016

Objectives: to examine the factorial validity and reliability of the Italian Version of the Dispositional Flow Scale-2, for use with Italian adults, young adults and adolescents

Authority: The Board is authorized to:

- Formulate rules or principles of ethics for adoption by the IAI psychologists;
- Evaluate research projects/activities carried out by IAI psychologists;
- Investigate allegations of unethical conduct of IAI psychologists;
- Resolve allegations of unethical conduct and/or recommend such action as is necessary to achieve the IAI ethical objectives.

Summary statement: This project has been reviewed and waived from Ethical Approval (see attached Ethical Issues Summary Table) **because it is a data collection study with no health care interventions.**

Prof. Andrea Gaggioli
Project Review Board (PRB)
I.R.C.C.S. Istituto Auxologico Italiano
20149, Milano

Tel. 02-619112892
Fax. 02-619112892

SUMMARY TABLE

The key principles of research ethics that have a **bearing on sharing or archiving personal data** are:

- a duty of confidentiality towards informants and participants;
- a duty to protect participants from harm, by not disclosing sensitive information;
- a duty to treat participants as intelligent beings, able to make their own decisions on how the information they provide can be used, shared and made public (through informed consent);
- a duty to inform participants how information and data obtained will be used, processed, shared, disposed of, prior to obtaining consent;
- a duty to wider society to make available resources produced by researchers with public funds (data sharing required by research funders).

If these principles are respected, no further ethical review is needed (**data collection study with no health care interventions)**.

	YES	CRITICAL
**Informed Consent**		
• Does the proposal involve children?		
• Does the proposal involve patients or persons not able to give consent?		
• Does the proposal involve adult healthy volunteers?	X	No[1]
• Does the proposal involve Human Genetic Material?		
• Does the proposal involve Human biological samples?		
• Does the proposal involve Human data collection?	X	No[2]
**Research on Human embryo/foetus**		
• Does the proposal involve Human Embryos?		
• Does the proposal involve Human Foetal Tissue / Cells?		
• Does the proposal involve Human Embryonic Stem Cells?		
**Privacy**		
• Does the proposal involve processing of genetic information or personal data (eg. health, sexual lifestyle, ethnicity, political opinion, religious or philosophical conviction)	X	No[3]
• Does the proposal involve tracking the location or observation of people?		
**Research on Animals**		
• Does the proposal involve research on animals?		
• Are those animals transgenic small laboratory animals?		
• Are those animals transgenic farm animals?		
• Are those animals cloned farm animals?		
• Are those animals non-human primates?		
**Research Involving Developing Countries**		
• Use of local resources (genetic, animal, plant etc)		
• Benefit to local community (capacity building i.e. access to healthcare, education etc)		
**Dual Use**		
• Research having direct military application		
• Research having the potential for terrorist abuse		
**ICT Implants**		
• Does the proposal involve clinical trials of ICT implants?		

[1] All participants formally agreed to participate in the study. No health care intervention was provided in the study (https://depts.washington.edu/bioethx/topics/consent.html).

[2] All participants formally agreed to participate in the study. No health care intervention was provided in the study (https://depts.washington.edu/bioethx/topics/consent.html).

[3] Participants were asked about their gender, age and level of studies. No sensitive data were collected. The applicants anonymised personal data before analysis and reporting
